# Methanol Vapor Retards Aging of PIM-1 Thin Film Composite Membranes in Storage

**DOI:** 10.1021/acsmacrolett.2c00568

**Published:** 2023-01-06

**Authors:** Ming Yu, Andrew B. Foster, Colin A. Scholes, Sandra E. Kentish, Peter M. Budd

**Affiliations:** † Department of Chemical Engineering, The University of Melbourne, Melbourne, VIC 3010, Australia; ‡ Department of Chemistry, School of Natural Sciences, The University of Manchester, M13 9PL Manchester, U.K.

## Abstract

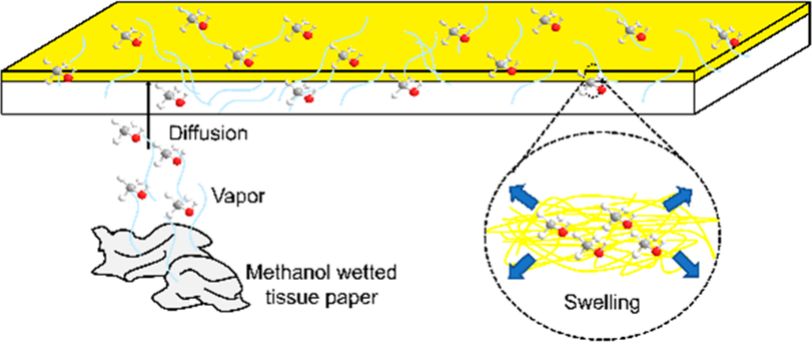

Physical aging of glassy polymers leads to a decrease in permeability over time when they are used in membranes. This hinders the industrial application of high free volume polymers, such as the archetypal polymer of intrinsic microporosity PIM-1, for membrane gas separation. In thin film composite (TFC) membranes, aging is much more rapid than in thicker self-standing membranes, as rearrangement within the thin active layer is relatively fast. Liquid alcohol treatment, which swells the membrane, is often used in the laboratory to rejuvenate aged self-standing membranes, but this is not easily applied on an industrial scale and is not suitable to refresh TFC membranes because of the risk of membrane delamination. In this work, it is demonstrated that a simple method of storage in an atmosphere of methanol vapor effectively retards physical aging of PIM-1 TFC membranes. The same method can also be utilized to refresh aged PIM-1 TFC membranes, and one-week methanol vapor storage is sufficient to recover most of the original CO_2_ permeance.

Glassy polymers tend to densify over time as the polymer chains relax toward a theoretical equilibrium state—a process termed physical aging.^1,2^ This has implications for their applications, for example, leading to a reduction in permeability over time when a glassy polymer is utilized in a gas separation membrane. The effects of physical aging are significant for high free volume polymers, such as polymers of intrinsic microporosity (PIMs), especially in the thin films applicable to industrially relevant membrane processes.^3^ Aging studies of polymeric membranes are often undertaken with intermittent testing, the membranes being stored between measurements. The conditions of storage may have a profound effect on the aging process. Here we demonstrate that a facile method of storing in methanol vapor can substantially retard aging of thin film composite membranes of the archetypal polymer of intrinsic microporosity PIM-1.

PIM-1 has received great attention since first reported in 2004.^4^ Because of the lack of conformational freedom, coupled with a highly contorted polymer backbone, PIM-1 membranes have high intrinsic free volume and thus high gas permeabilities.^5^ Furthermore, high chemical, thermal, and mechanical stability, solution processability, and reasonable CO_2_ selectivities over competing gases make PIM-1 a promising candidate for industrial application.^6^ However, like other high free volume glassy polymers, it is prone to physical aging, leading to a significant reduction in gas permeability over time.^2,7^ Physical aging is thickness-dependent and happens more rapidly in a thinner film,^7,8^ which impedes the application of PIM-1 based thin film composite (TFC) membranes, which have selective layer thickness below a couple of micrometers and thus permeances tens to hundreds of times higher than self-standing membranes.^9^

A number of factors may influence physical aging. High intrachain rigidity, although attractive for creating high free volume and a better molecular sieving effect, can lead to faster aging.^10^ Polymer topology also accounts for different aging behavior, with a branched PIM-1 structure maintaining a better separation performance over time than a disubstituted structure.^11,12^ Mixed matrix membranes (MMMs) offer a way to retard aging, and various types of fillers have been investigated, including carbon nanotubes,^13^ graphene oxide (GO) and its derivatives,^14−16^ 2D nanosheets,^17^ cross-linked polymer,^9,18^ and metal–organic frameworks (MOFs).^19,20^ Membrane post-treatments such as thermal cross-linking,^21^ ultraviolet treatment,^22^ and supercritical CO_2_ treatment^23^ can also affect the rate of physical aging.

Physical aging is reversible, and lost free volume can be recovered by immersing aged membranes in a liquid alcohol (methanol, ethanol, etc.).^8,24^ Liquid alcohol can swell a membrane,^25^ enhance the mobility of polymer chains,^26^ and “reset” the membrane after aging. Such treatment may also displace any entrapped solvents and other contaminants from membrane preparation and operation^10^ and result in more free volume than as-cast membranes.^27^ However, this method is not applicable at the industrial scale, where membranes are integrated into modules that are not designed for contacting with liquid. In addition, liquid alcohol immersion cannot generally be applied to TFC membranes due to the risk of membrane delamination and the possibility of affecting the porosity of the support. A vapor treatment offers a feasible alternative to liquid alcohol immersion. Liu et al.^19^ applied a methanol vapor treatment to aged PIM-1 TFC membranes, and the refreshed membranes recovered 40% of initial CO_2_ permeance. Almansour et al.^28^ developed a methanol vapor treatment method to rejuvenate PIM-1 thick films. An 8 h treatment was enough to refresh an aged PIM-1 membrane back to a similar gas separation performance as day 1, but vacuum and heat were required.

There are few studies in which membrane aging has been investigated under continuous, rather than intermittent, operation. Sekizkardes et al.^29^ subjected a PIM-1/polyphosphazene blend membrane to real postcombustion flue gas (approximately 80% N_2_, 10% CO_2_, 9% O_2_, 10 ppm of NO_2_, and 1.3 ppm of SO_2_, with humidity) over a 556 h period and saw little change in performance under continuous operation. This shows that PIM-based membranes are capable of stable performance under operating conditions and suggests they are promising materials for use as reverse-selective membranes. Pilnáček et al.^30^ studied the permeation of methanol vapor at an activity of 0.2 through PIM-1 and PIM-EA-TB membranes, comparing a continuous 650 h experiment with a series of short-term 6 h experiments. The permanent presence of mobility-enhancing methanol in the membranes under these conditions led to faster aging in continuous than in intermittent (momentary) mode.

While long-term testing under continuous operation is the ideal way to assess membrane performance, many laboratories are unable to commit equipment to a single experiment for an extended period and therefore undertake intermittent testing. The present work aimed to establish whether aging during storage could be mitigated by keeping membranes in an atmosphere of methanol vapor that would be adsorbed and keep the polymer in a swollen state. A simple methanol vapor storage method was developed to gently offset the aging effect of PIM-1 TFC membranes at room temperature and ambient pressure. PIM-1 TFC membranes were prepared by a kiss-coating method using various solvents, solution concentrations, and PIM-1 topologies. As demonstrated below, gas separation performance (e.g., CO_2_ permeance, CO_2_/N_2_, and CO_2_/CH_4_ selectivity) was stable over 28 days aging when stored in a methanol vapor atmosphere. The same methodology could also be applied to refresh aged PIM-1 TFC membranes.

As shown in Figure 1, PIM-1 TFC membranes were stored in a sealed ziplock bag, accompanied by an open plastic bag with methanol wetted tissue papers in it, which allowed slow evaporation of methanol to create a methanol vapor atmosphere. Methanol vapor gently diffused into the PIM-1 TFC membranes, swelled the polymer structure, and mitigated physical aging without causing any membrane delamination. Before any gas permeation test, TFC membranes were placed in a N_2_ cabinet overnight to remove methanol vapor. More details regarding the storage method are provided in the Supporting Information.

**Figure 1:**
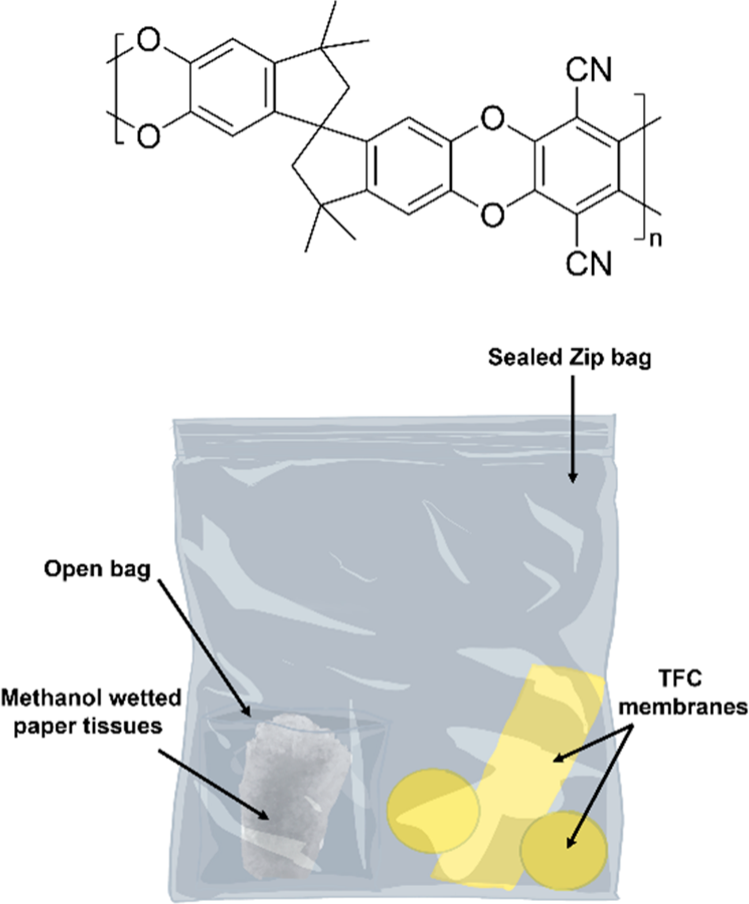
Chemical structure of PIM-1 and method of PIM-1 TFC membrane storage under a methanol vapor environment.

Three samples of PIM-1 were used in this work: two branched (B1, B2) and one disubstituted (D1) (see Table S1). PIM-1 TFC membranes were kiss-coated from 0.6% to 3% w/v solutions of PIM-1 in chloroform (CHCl_3_) and tetrahydrofuran (THF) directly onto polyacrylonitrile (PAN) ultrafiltration supports without applying a gutter layer. Two different batches of support were used (PAN_01 and PAN_02) which exhibited differences in surface texture, pore size, and porosity by scanning electron microscopy (see Figure S6). Gas separation performance at day 1 is shown in Figure 2. For PIM-1 TFC membranes prepared from THF solutions and PAN_01 support, solution viscosity decreased as the coating concentration decreased, allowing the coating solution to soak into the support more easily. Though a thinner active layer should be obtained,^31^ the mixed PIM-1/PAN interface caused additional gas transport resistance, which resulted in decreased gas permeance. The selectivity of both CO_2_/N_2_ and CO_2_/CH_4_ increased with decreasing coating solution concentration, which might be attributed to fewer defects because of better interaction between PIM-1 and the support due to solution soaking.^32^ The same trend of decreasing permeance with decreasing coating solution concentration was observed for PIM-1 membranes prepared from CHCl_3_ solutions using the same support, but the selectivity change was small. In addition, for the same coating solution concentration, the CO_2_ permeance of PIM-1 TFC membranes prepared from CHCl_3_ was higher than that prepared from THF. THF has a strong affinity toward water,^33^ which could lead to water entrapment in the membranes, affecting gas separation performance.^34^ PAN_02 was also used to prepare TFC membranes from THF. As the coating solution concentration decreased, the CO_2_ permeance went through a maximum, with selectivity changing in the opposite direction. PAN_01, with its smoother surface texture and higher pore density, appears to facilitate greater ingression of polymer into the PAN support during the kiss-coating process. However, PAN_02 has both a rougher surface and a lower pore density, which seems to favor the creation of a distinct surface layer. At 3% and 1.5% solution concentration, the penetration effect was small, and the active layer dominated gas transport resistance. When the concentration further decreased to 0.7%, solution soaking into the support and the sealing of coating defects may account for the decreased permeance and increased selectivity.

**Figure 2:**
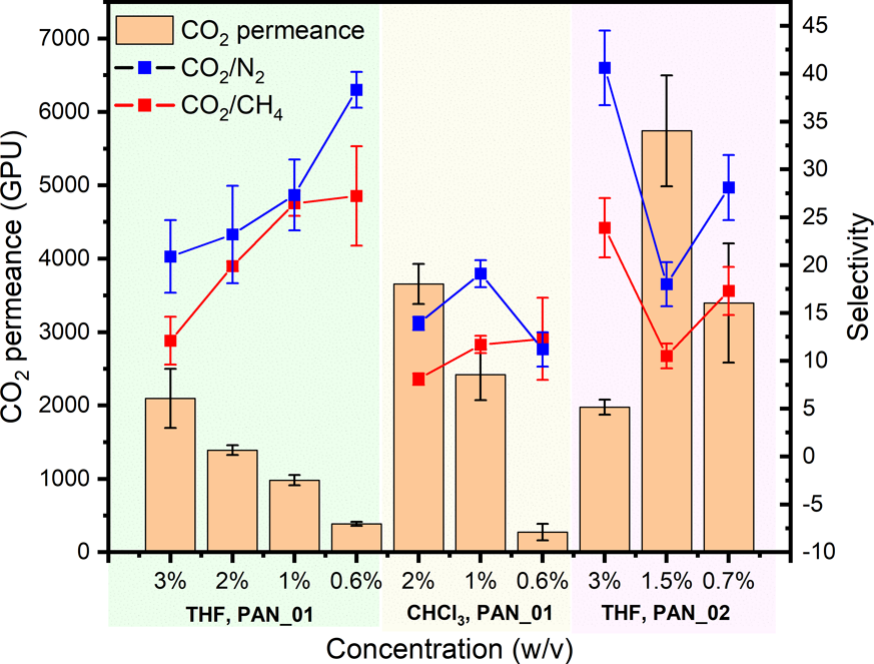
PIM-1 TFC membrane gas separation performance (CO_2_ permeance, selectivity of CO_2_/N_2_ and CO_2_/CH_4_) at day 1. Membranes were kiss-coated from both THF and CHCl_3_ solutions with various concentrations (0.6—3%, w/v) on different batches of PAN support. PIM-1 B1 was used on PAN_01, and B2 was used on PAN_02.

Normal aging and methanol vapor retarded aging data are shown in Figure 3 and summarized in Table S2. The CO_2_ permeance of PIM-1 TFC membranes stored under ambient conditions between measurements fell rapidly in the first week and more slowly thereafter.^9^ Membranes lost more than 50% CO_2_ permeance during 4 weeks of aging. However, TFC membranes stored in a methanol vapor environment actually showed an increase in CO_2_ permeance. Because of its low boiling point and high volatility, liquid methanol easily vaporizes at room temperature and atmospheric pressure. Once TFC membranes are placed in a methanol vapor atmosphere, methanol vapor is expected to gently diffuse into the membranes and swell the polymer,^10^ creating additional free volume, leading to increased gas permeance but decreased gas selectivity.^24,28^ Membrane gas separation performance then tended to be stable. Our results showed that up to 4-week methanol vapor storage did not result in any membrane delamination. The adsorbed methanol vapor easily diffused out of the membranes when placed in a nitrogen cabinet overnight.

**Figure 3:**
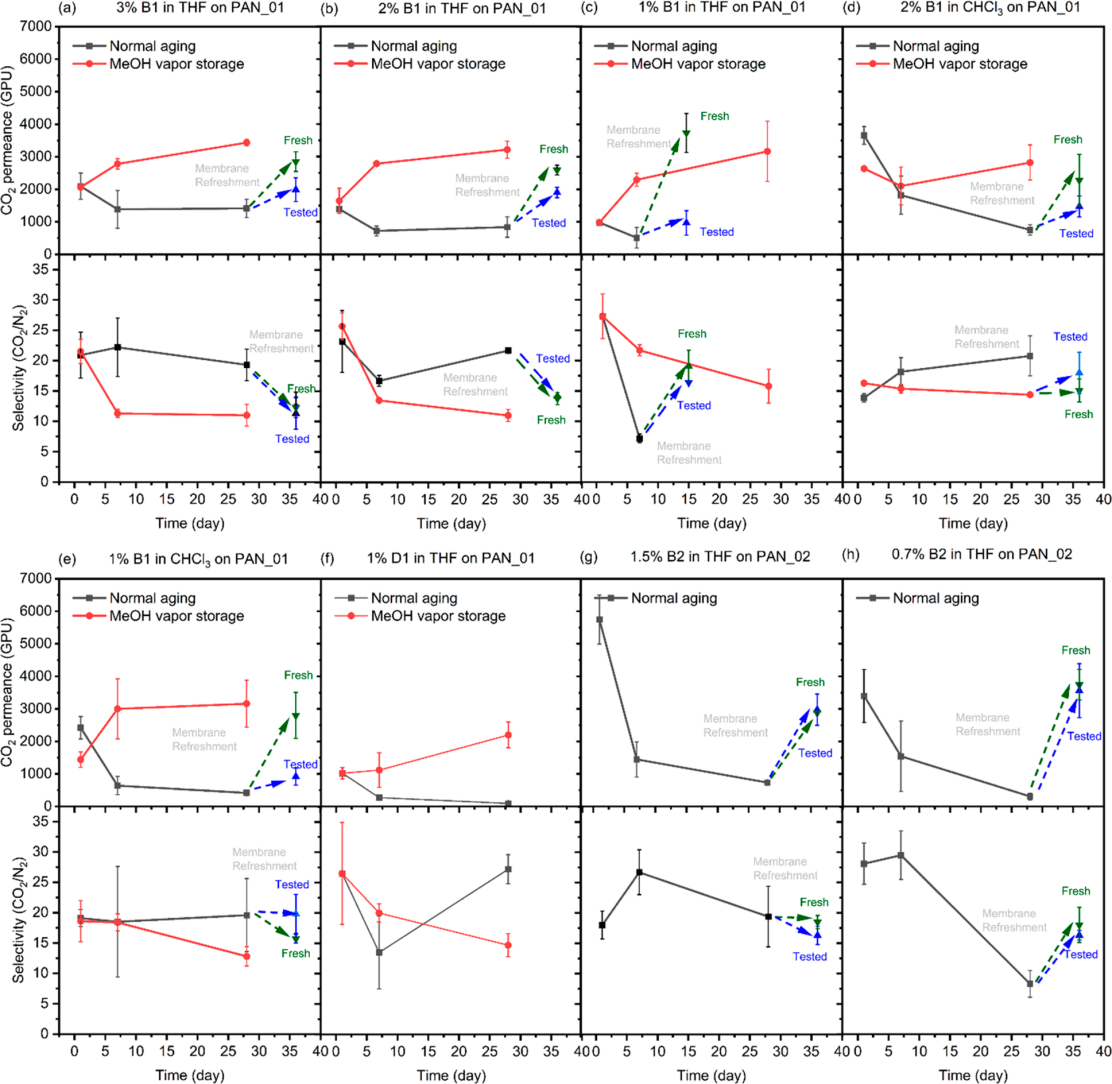
Changes in CO_2_ permeance and CO_2_/N_2_ selectivity on aging of PIM-1 TFC membranes with normal storage and methanol vapor storage. TFC membranes were prepared using branched PIM-1 B1 from (a) 3%, (b) 2%, and (c) 1% THF solutions and (d) 2% and (e) 1% CHCl_3_ solutions and (f) using disubstituted PIM-1 D1 from 1% THF solution on PAN_01 support. TFC membranes were prepared using branched PIM-1 B2 from (g) 1.5% and (h) 0.7% THF solutions on PAN_02 support. After 28 days of the normal aging process, TFC membranes were further refreshed by storing in a methanol vapor atmosphere for 7 days. TFC membranes that had been tested previously and then retested are labeled as tested (blue), and TFC membranes that had not been tested previously are labeled as fresh (green).

For those TFC membranes prepared at low concentrations (≤1%) from THF with significant solution penetration, the CO_2_ permeance dropped rapidly along with a significant selectivity decrease upon normal storage of 7 days. Similar aging behavior was observed as with other samples upon longer term storage.^12^ Such an effect could be avoided through methanol vapor storage. Moreover, those aged TFC membranes could also be rejuvenated by the same method, storing in a methanol vapor environment for 7 days. Both fresh samples (never characterized for gas separation performance) and tested samples (characterized at least once after coating) were used for membrane refreshment. As seen in Figure 3, the CO_2_ permeance was recovered in the refreshed TFC membranes due to the swelling effect of methanol vapor.^28^ Most of the fresh samples showed a fully recovered separation performance, identical with samples stored in methanol vapor. However, some of the tested samples only gave a partial recovery, indicating that the testing process and the presence of other gases may affect the membrane. Nevertheless, the rejuvenated performance of tested samples was still comparable with the untreated performance at day 1.

In conclusion, we propose a facile methanol vapor storage method to counteract the effect of physical aging on PIM-1 TFC membranes. This method is applicable at room temperate and atmospheric pressure. The same method can also be used to refresh aged TFC membranes. Refreshed membranes showed better gas separation performance than before treatment and at least partially recovered the performance of freshly prepared samples. In industry, a similar approach could be used for membrane modules to maintain freshness when stored before use. A small amount of methanol could be introduced to maintain a methanol vapor pressure in the module, similar to the way glycerin is used for preservation of ultrafiltration membranes.^35^ Future work will focus on optimizing the methodology for storing and rejuvenating TFC membranes.

## Data Availability

Data supporting this study are available within the Article and the Supporting Information.

## Supporting Information Available

Materials; monomer purification; PIM-1 synthesis and purification; PIM-1 characterization; PAN support characterization; PIM-1 thin film composite membrane preparation; methanol vapor storage; gas permeation tests; temperature profile and torque profile of PIM-1 synthesis; thin film coating setup; PIM-1 characterization data; ^1^H NMR spectra of PIM-1 polymers; SEM images of PAN supports; gas permeation data (PDF)
