# Supplementary material for: Methanol Vapor Retards Aging of PIM-1 Thin Film Composite Membranes in Storage
Source: ACS Macro Lett. 2023 Jan 6;12(1):113–7. doi: 10.1021/acsmacrolett.2c00568 (PMC9850912; doi:10.1021/acsmacrolett.2c00568)
Supplement: Supplementary file 1 — mz2c00568_si_001.pdf [file mz2c00568_si_001.pdf]

Supporting information

Methanol Vapor Retards Aging of PIM-1 Thin Film Composite Membranes in Storage

Ming Yu [1,2], Andrew B. Foster [2], Colin A. Scholes *[1], Sandra E. Kentish [1], and Peter M. Budd *[2]

1 Department of Chemical Engineering, The University of Melbourne, Melbourne, VIC. 3010, Australia.

2 Department of Chemistry, School of Natural Sciences, The University of Manchester, M13 9PL Manchester, U.K.

**Corresponding authors:* Peter.Budd@manchester.ac.uk, cascho@unimelb.edu.au

Contents

Materials.....2

Monomer purification.....2

PIM-1 synthesis and purification.....2

PIM-1 characterization.....3

PAN support characterization.....4

PIM-1 thin film composite membrane preparation.....4

Methanol vapor storage.....4

Gas permeation tests.....5

Figure S1. Temperature profile and torque profile of PIM-1 synthesis.....6

Figure S2. Thin film coating setup with a steel coater connected to a motor, steel bucket for containing coating solution, and glass plates for supporting the bucket to control the contact between PAN support and solution. 6

Table S1. PIM-1 polymers produced from step growth polymerizations in dilute solvent mixtures, and their characterization by multi-detector GPC analysis. 7

Figure S3. Chemical structures of di-substituted PIM-1 and a branch point in PIM-1 7

Figure S4. ^{1}H NMR spectrum of branched PIM-1 polymer B1. Two minor shoulder peaks at 6.2 and 6.6 ppm suggest branched structure. 8

Figure S5. ^{1}H NMR spectrum of branched, PIM-1 polymer B2. Two minor shoulder peaks at 6.2 and 6.6 ppm suggest branched structure. 8

Figure S6. ^{1}H NMR spectrum of disubstituted, PIM-1 polymer D1. 9

Figure S7. Surface SEM images of ultrafiltration supports PAN_01 and PAN_02. PAN_01 has higher surface porosity, higher pore size and less texture than PAN_02, which leads to it being more prone to solution soaking..9

Table S2. TFC membrane normal aging, refreshment, and methanol vapor storage data of materials coated on PAN_01 support (data are averaged for at least two coupons, with standard deviation). 10

Table S3. TFC membrane normal aging, refreshment data of materials coated on PAN_02 support (data are averaged for at least two coupons, with standard deviation). 11

Materials

Monomer 5,5´,6,6´-tetrahydroxy-3,3,3´,3´-tetramethyl-1,1´-spirobisindane (TTSBI, 97%) was purchased from Alfa Aesar and purified further before use as described below. Monomer tetrafluoroterephthalonitrile (TFTPN, >99%) was purchased from Fluorochem. Potassium carbonate (K_2CO_3, anhydrous, ≥ 99.5%), tetrahydrofuran (THF, analytical reagent grade, ≥ 99.8%) were purchased

from Fisher Scientific. Chloroform (HPLC, ≥ 99.8%), ethyl acetate (GC, ≥ 99.5%), hexane (HPLC, ≥ 97%), toluene (ACS reagent, ≥ 99.7%), N,N-dimethylacetamide (DMAc, anhydrous, 99.8%), acetone (for analysis), methanol (ACS reagent, ≥ 99.8%), 1,4-dioxane (anhydrous, 99.8%), chloroform-d (99.8 atom %D) and glass wool were purchased from Sigma-Aldrich. Polyacrylonitrile (PAN) ultrafiltration membrane support was purchased from SolSep BV (The Netherlands). Two different batches of UF010104 support were used, PAN_01 is batch G and PAN_02 is batch 1501.

Monomer purification

TTSBI monomer required further purification before use. 20 g TTSBI was refluxed in 333 ml ethyl acetate at 90 °C under a nitrogen environment for 2 h. 333 ml hexane was added then the mixture was left to reflux at 90 °C for 10 min. The mixture was then cooled down in ice for 3 h before collection by filtration. The purified monomer was dried under vacuum for at least one day before use.

TFTPN monomer was used as received, but required vacuum drying for at least one day before use.

PIM-1 synthesis and purification

PIM-1 was synthesized based on a high temperature synthesis method.[1] A 500 ml three-neck round bottom flask was fitted with an N_2 flow inlet, an open-end coil condenser and an overhead stirrer (Heidolph Instruments Hei-TORQUE Expert 100, Germany) and placed in a heating block (Asynt, UK) on a hot plate (IKA, UK). 17.03 g (50 mmol) TTSBI, 10 g (50 mmol) TFTPN and 20.73 g (150 mmol) K_2CO_3 were added to the flask. Then 180 ml solvent mixture (DMAc and toluene mixed at a ratio of 2:1) was added into the flask, and the overhead stirrer (set at 250 rpm), hot plate (set at 160 °C) and N_2 flow were turned on immediately. The stir speed was increased gradually to ensure uniform mixing as the viscosity increased over time. Two batches of extra solvent mixture (30 ml) were added into the flask during the

reaction **(Table S1)**. The polymerization was run for 30-40 min and was quenched through pouring the solution into excess of methanol.

Crude PIM-1 polymer was collected by vacuum filtration and redissolved in 700 ml chloroform. PIM-1 was then reprecipitated by slowly pouring the solution into an excess of methanol. Vacuum filtered PIM-1 was refluxed in deionized water overnight and recovered by vacuum filtration again. Then PIM-1 was immersed in a minimum amount of 1,4-dioxane (just enough to cover the polymer) for 15 min, followed by washing with copious amounts of acetone and methanol, and then immersed in methanol overnight. Finally vacuum filtration recovered PIM-1 was dried in a vacuum oven at 120 ℃ for three days before use.

It should be noted that the heating rate can affect PIM-1 topology. The heating rate was controlled through using different sized heating blocks, with a larger heating block giving a slower heating rate and a smaller heating block giving a faster heating rate. A slower heating rate from room temperature predominately results in a branched structure and a faster heating rate produces a di-substituted structure,[1] as indicated by the ^{1}H NMR spectra (**Figure S2-S4**).

PIM-1 characterization

Proton nuclear magnetic resonance (^{1}H NMR) spectra were collected from 25 mg ml^{-1} PIM-1 solutions in chloroform-d using a Bruker Avance II 500 MHz instrument. Two main peaks, appearing at 6.4 ppm and 6.7 ppm, are from the aromatic protons due to the spiro-center of the TTSBI monomer. A slower heating rate of polymerization gives more mono-substituted connections which lead to a branched structure. The branched structure is reflected in the ^{1}H NMR spectrum with two minor peak shoulders at 6.2 and 6.6

ppm adjacent to the main aromatic proton peaks, which are attributed to the aromatic protons adjacent to the branching -OH point (**Figure S2** and **Figure S3**).[2]

The number-average molar mass M_n, weight-average molar mass M_w, and dispersity $Đ$ of the PIM-1 samples were determined using multi-detector gel permeation chromatography (GPC). PIM-1 polymers were prepared as 1 mg ml^{-1} solutions in chloroform followed by filtration using a polytetrafluoroethylene (PTFE) membrane filter (0.45 μm, Fisherbrand). Samples were characterized using a Viscotek VE2001 SEC solvent / sample module with two PL Mixed B columns and a Viscotek TDA 302 triple detector array (refractive index, light scattering, viscosity detectors. The column temperature was 35 ℃. The system was calibrated using a 110 kg/mol polystyrene standard and data was analyzed using OmniSEC software.

PAN support characterization

Two batches of ultrafiltration support, PAN_01 and PAN_02, were characterized by Quanta 250 FEG-SEM (FEI, USA) scanning electron microscope. Before imaging, samples were sputter-coated with Pt (108 Auto, Cressington Scientific Instruments, UK). This work was carried out by Dr. Monica Alberto, University of Manchester.

PIM-1 thin film composite membrane preparation

PIM-1 thin film composite (TFC) membranes were prepared using a roller-coater (**Figure S2**). PAN ultrafiltration support was cut into a rectangular sheet with dimensions of 4.5 cm × 10 cm. The support was then attached to the roller wheel with edges sealed with aluminum tape to prevent any solution soaking to the bottom of the support. The roller-coater was connected to a programmable DC power supply motor (RS-3005P, RS PRO, UK) with voltage of 15 V and current reading around 0.14 A. PIM-1

solution was prepared as 0.6 ~ 3% w/v in either chloroform or THF and was poured into a steel bucket below the coater with height adjusted by glass plates underneath. After coating, the sheets were peeled off from the roller and placed in a nitrogen atmosphere storage cabinet[2] at room temperature overnight before any testing. However, it is difficult to achieve perfect control of uniform coating using a small, laboratory roller-coater. For low concentration coating, where significant solution penetration is prone to happen, it can be even harder to achieve good control. This can lead to variation in the performance, especially upon aging.

Methanol vapor storage

A zip bag (8 cm × 15 cm) was used as a container. Five sheets of paper tissue (Kimwipe), wetted by 10 ml methanol, were placed in an opened zip bag, and 3~4 PIM TFC sheets (4 cm × 10 cm) were stored with it. In such a sealed bag, equilibrium is expected to be reached between liquid methanol and methanol vapor, and the vapor pressure would be the saturated vapor pressure of methanol (0.13 bar at a room temperature of 20 °C). The key point is to ensure that the paper tissue remains wet during the storage period and the liquid-gas equilibrium is maintained for a saturated methanol vapor environment.

Gas permeation tests

Gas permeance tests were performed at room temperature by the standard variable volume method,[3] and N_2, CH_4 and CO_2 pure gases were used in that sequence. Upstream gauge pressure was maintained at 35 psi (2.41 bar) with downstream at atmospheric pressure. PIM-1 TFC membranes were cut into circular coupons to fit the testing rig with active permeation area of 2.84 cm^2. Before collecting any data, the membranes were pre-conditioned under the testing pressure for 5 min for each gas. Then the time for a

specific volume of gas to permeate through the membrane was recorded. Membrane permeance was calculated based on the following equation:

$$K = \frac{Q}{tA(p_1 - p_2)} \times 10^6 \quad (1)$$

In this equation, K is the gas permeance (GPU, 1 GPU = 10^{-6} cm^3 [STP] cm^{-2} s^{-1} cmHg^{-1} = 3.348×10^{-10} mol m^{-2} s^{-1} Pa^{-1}), t is the permeation time (s), Q is the volume of gas that permeates through the membrane during the permeation time (cm^3, corrected to STP [0 °C, 1 atm]), A is the active permeation area (cm^2), and p_1 and p_2 are the pressure in the membrane feed side and permeate side (cmHg), respectively.

The membrane ideal gas selectivity was calculated as the ratio of gas permeances by the following equation:

$$\alpha_{CO_2/x} = \frac{K_{CO_2}}{K_x} \quad (2)$$

where x is either N_2 or CH_4. TFC membranes, with both normal storage and methanol vapor storage conditions, were characterized to track physical aging across a period of 28 days. Aged TFC membranes were further refreshed by storing them under a methanol vapor atmosphere for 7 days. At least two membranes of each sample were tested for reproducibility, and the average with standard deviation is reported.

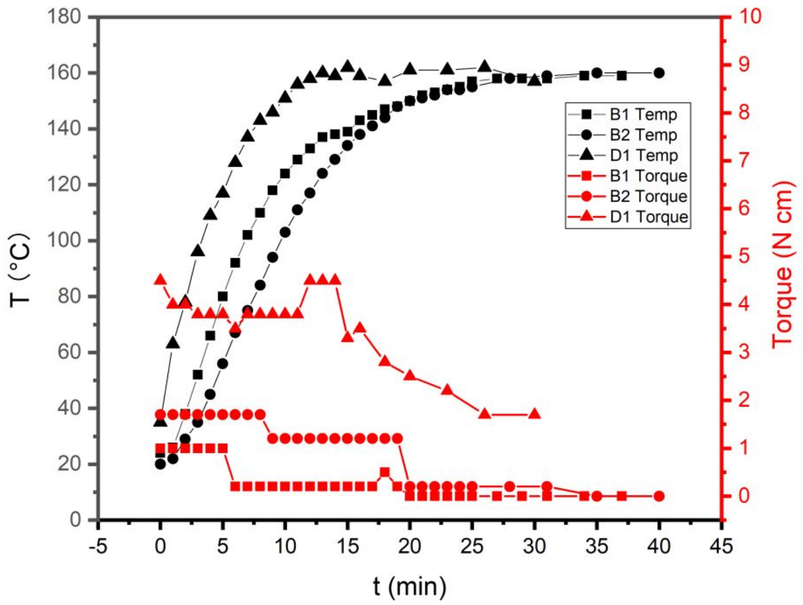

Figure S1. Temperature profile and torque profile of PIM-1 synthesis.

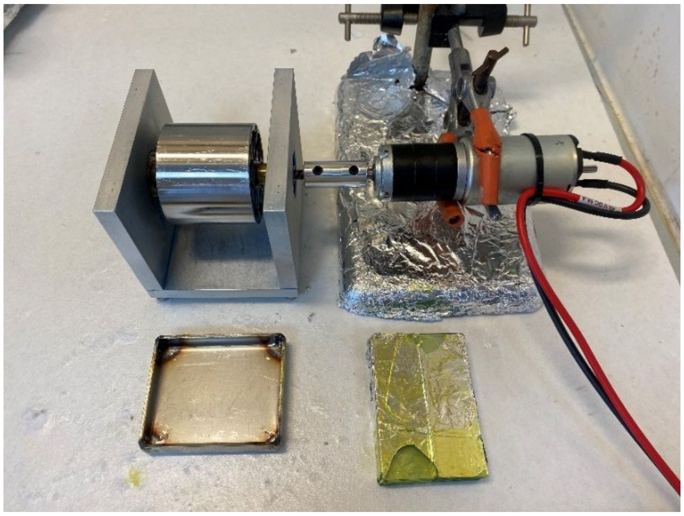

Figure S2. Thin film coating setup with a steel coater connected to a motor, steel bucket for containing coating solution, and glass plates for supporting the bucket to control the contact between PAN support and solution.

Table S1. PIM-1 polymers produced from step growth polymerizations in dilute solvent mixtures, and their characterization by multi-detector GPC analysis.

PIM-1	Structure	Temp.[1] Set/Av. (°C)	Time (min)	Times of addition of extra solvent (min)	Yield (%)	M_w[2] (g mol^{-1})	M_n[3] (g mol^{-1})	Đ[4]	Intrinsic Viscosity (cm^3 g^{-1})	Hydrodynamic Radius (nm)
**B1**	Branched	160/130	37	9 & 14	96	106,140	45,720	2.32	34	7.8
**B2**	Branched	160/126	40	15 & 22	95	134,000	54,110	2.48	37.2	8.6
**D1**	Di-substituted	160/143	30	8 &11	85	169,410	98,070	1.73	44.2	10

[1]*Set/average polymerization temperature.* [2]*Weight-average molar mass.* [3]*Number-average molar mass.* [4]*Dispersity =* M_w/M_n.

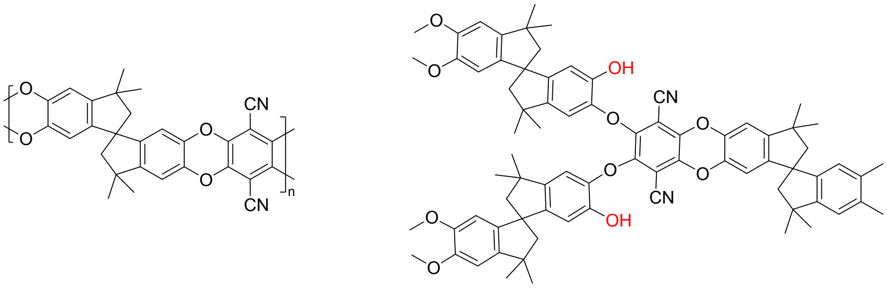

Disubstituted Branch Point

Figure S3. Chemical structures of di-substituted PIM-1 and a branch point in PIM-1.

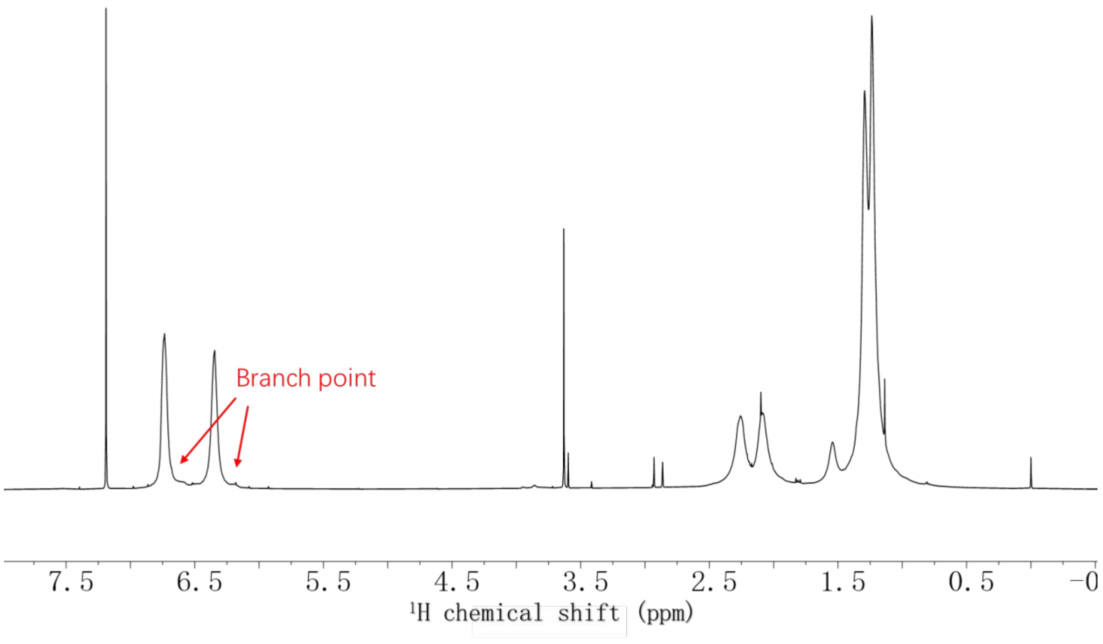

Figure S4. ^{1}H NMR spectrum of branched PIM-1 polymer B1. Two minor shoulder peaks at 6.2 and 6.6 ppm suggest branched structure.

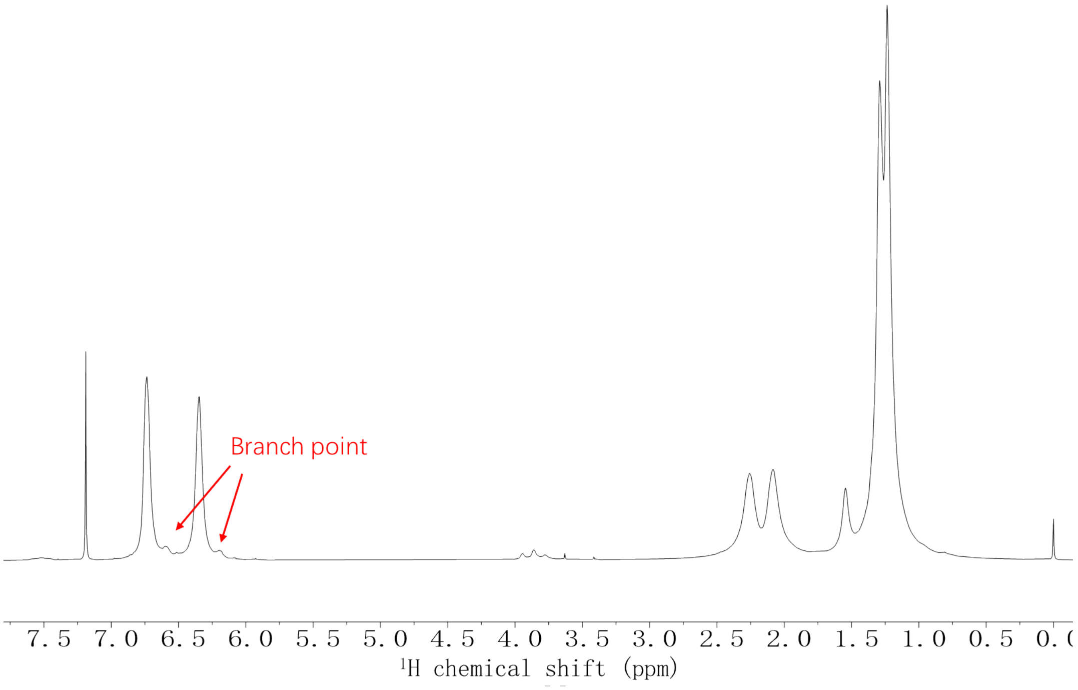

Figure S5. ^{1}H NMR spectrum of branched, PIM-1 polymer B2. Two minor shoulder peaks at 6.2 and 6.6 ppm suggest branched structure.

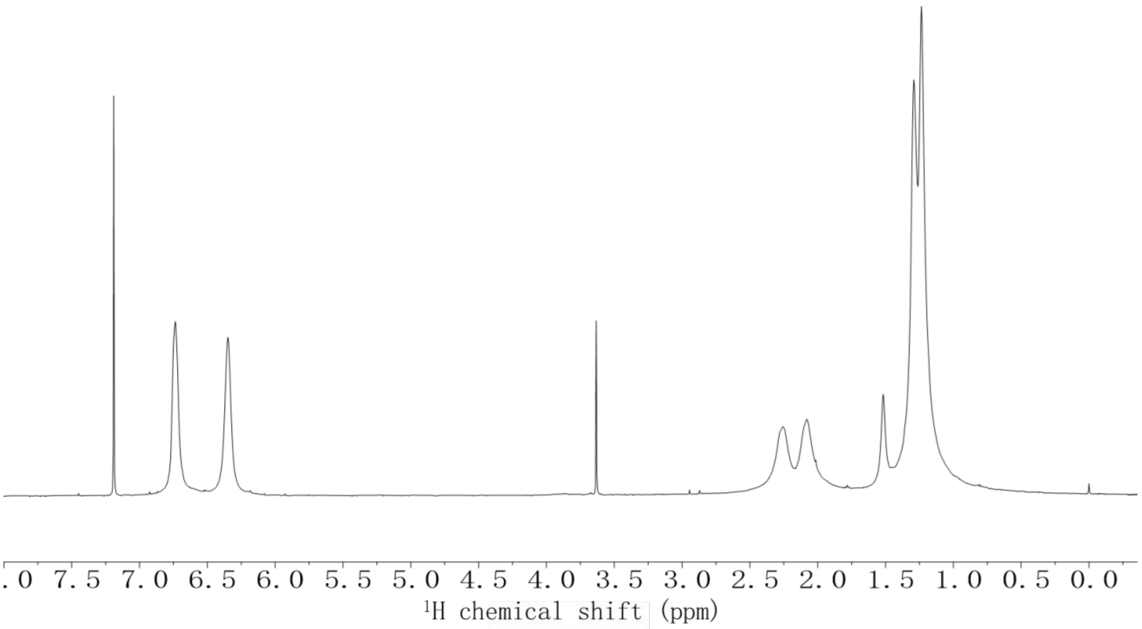

Figure S6. ^{1}H NMR spectrum of disubstituted, PIM-1 polymer D1.

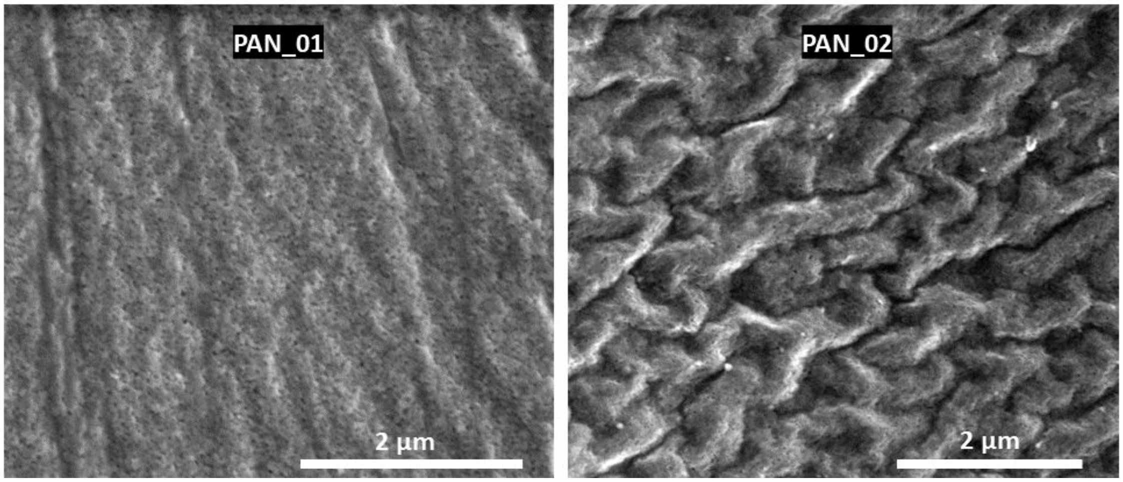

Figure S7. Surface SEM images of ultrafiltration supports PAN_01 and PAN_02. PAN_01 has higher surface porosity, higher pore size and less texture than PAN_02, which leads to it being more prone to solution soaking.

Table S2. TFC membrane normal aging, refreshment, and methanol vapor storage data of materials coated on PAN_01 support (data are averaged for at least two coupons, with standard deviation).

Sample[1]	Time[4] (days)	Normal aging[2] Permeance CO_2 (GPU)	Normal aging[2] Selectivity CO_2/N_2	Normal aging[2] Selectivity CO_2/CH_4	MeOH vapor storage[3] Permeance CO_2 (GPU)	MeOH vapor storage[3] Selectivity CO_2/N_2	MeOH vapor storage[3] Selectivity CO_2/CH_4
3% B1 in THF on PAN_01	1	2100±400	21±3.8	12±2.5	2100±6	22±2	12±3.7
	7	1400±580	22±4.8	15±4.5	2800±170	11±0.7	6.2±0.4
	28	1400±280	19±2.6	12±1.8	3400±95	11±1.8	5.6±0.4
	36_tested	2000±360	11±2.6	6.2±1.2			
	36_fresh	2900±302	12±1.7	6.2±0.1	2300 460	13 1.6	7.8 0.4[5]
2% B1 in THF on PAN_01	1	1400±67	23±5.1	20±0.5	1600±390	26±2.2	17±3.6
	7	720±150	17±0.9	20±1.5	2800±80	14±0.2	7.5±0.1
	28	840±310	22±0.4	17±1.6	3200±260	11±1	6.2±0.5
	36_tested	1900±160	14±1.6	8±0.4			
	36_fresh	2600±150	14±1	7.7±0.6	1200 360	11 0.4	6.9 0.2[5]
1% B1 in THF on PAN_01	1	980±70	27±3.7	27±1.3	980±70	27±3.7	27±1.3
	7	510±320	7.2±0.7	5±0	2300±200	22±0.9	13±0.7
	28				3200±930	16±2.8	9.1±1.7
	15_tested	970±370	19±2.6	13±1.2			
	15_fresh	3700±600	16±0.2	9.2±0.3			
0.6% B1 in THF on PAN_01	1	390±29	38±1.9	27±5.2			
	7	130±29	9.2±3.7	8.8±2.9			
	15_tested	590±160	13±0.5	12±2.9			
	15_fresh	1800±80	24±1.6	15±1			
2% B1 in $CHCl_3$ on PAN_01	1	3700±270	14±0.7	8.1±0.6	2600±13	16±0.3	9.6±0.1
	7	1800±580	18±2.3	11±1.8	2100±580	15±0.7	8.6±0.5
	28	750±160	21±3.3	15±2.3	2800±540	14±0.3	7.3±0.5
	36_tested	1500±324	18±3.4	9.4±0.7			
	36_fresh	2300±790	15±1.9	8.1±1			
1% B1 in $CHCl_3$ on PAN_01	1	2400±350	19±1.4	12±0.9	1400±240	19±3.4	12±2.9
	7	640±280	19±9.1	19±1.7	3000±920	18±1.4	11±1
	28	420±20	20±6	15±2.2	3200±720	13±1.6	7.6±0.9
	36_tested	920±260	20±3.2	13±0.6			
	36_fresh	2800±710	16±0.7	9.2±0.6			
0.6% B1 in $CHCl_3$ on PAN_01	1	280±110	11±1.8	12±4.4			

1% D1 in THF on PAN_01	1	1000±170	27±8.4	20±3.6	1000±170	27±8.4	20±3.6
	7	270±59	14±6	9.6±6.9	1100±533	20±1.5	14±0.7
	28	96±8	27±2.4	20±2.2	2200±400	15±1.9	8.8±1.1

[1]Samples are named as concentration (w/v), polymer (type) in solvent on support (type). [2]TFC membranes were kept in sealed zip bag as normal storage condition. [3]TFC membranes were kept under a methanol vapor atmosphere in a sealed zip bag to retard membrane aging. [4]After normal aging process, PIM-1 TFC membranes were further rejuvenated through storing in a methanol vapor environment for 7 days followed by storing in a N_2 cabinet for another day prior to gas permeation test. Membranes are named as Time_tested or fresh, with TFC membranes that had been tested previously and then retested labeled as tested and TFC membranes that had not been tested previously labeled as fresh. [5]After 28 days of methanol vapor storage, TFC membranes were kept under normal condition for 8 days to track physical aging, and fresh samples were tested.

Table S3. TFC membrane normal aging, refreshment data of materials coated on PAN_02 support (data are averaged for at least two coupons, with standard deviation).

Sample	Time (days)	Normal aging: Permeance CO_2 (GPU)	Selectivity CO_2/N_2	Selectivity CO_2/CH_4
3% B2 in THF on PAN_02	1	2000±100	41±3.9	24±3
	6	1200±90	35±2.4	27±2
	60	450±280	20±6.7	18±0.7
1.5% B2 in THF on PAN_02	1	5700±760	18±2.3	11±1.3
	7	1400±540	27±3.7	21±3.5
	28	740±76	19±5	20±1.4
	36_tested	3000±480	16±1.5	8.8±0.7
	36_fresh	3000±540	19±1.1	10±0.6
0.7% B2 in THF on PAN_02	1	3400±800	28±3.4	17±2.5
	7	1500±1100	30±4	23±0.9
	28	310±100	8.3±2.2	9.5±1.1
	36_tested	3600±830	16±0.8	9.3±0.1
	36_fresh	3700±470	18±2.9	10±1.7

References

(1) Foster, A. B.; Beal, J. L.; Tamaddondar, M.; Luque-Alled, J. M.; Robertson, B.; Mathias, M.; Gorgojo, P.; Budd, P. M. Importance of small loops within PIM-1 topology on gas separation selectivity in thin film composite membranes. *J. Mater. Chem. A* **2021**, *9*, 21807-21823. DOI: 10.1039/d1ta03712a.

(2) Foster, A. B.; Tamaddondar, M.; Luque-Alled, J. M.; Harrison, W. J.; Li, Z.; Gorgojo, P.; Budd, P. M. Understanding the Topology of the Polymer of Intrinsic Microporosity PIM-1: Cyclics, Tadpoles, and Network Structures and Their Impact on Membrane Performance. *Macromolecules* **2020**, *53*, 569-583. DOI: 10.1021/acs.macromol.9b02185.

(3) Devarajan, A.; Asuquo, E. D.; Ahmad, M. Z.; Foster, A. B.; Budd, P. M. Influence of Polymer Topology on Gas Separation Membrane Performance of the Polymer of Intrinsic Microporosity PIM-Py. *ACS Appl. Polym. Mater.* **2021**, *3*, 3485-3495. DOI: 10.1021/acsapm.1c00415.
